# pH-Responsive PVA/BC-*f*-GO Dressing Materials for Burn and Chronic Wound Healing with Curcumin Release Kinetics

**DOI:** 10.3390/polym14101949

**Published:** 2022-05-11

**Authors:** Wafa Shamsan Al-Arjan, Muhammad Umar Aslam Khan, Hayfa Habes Almutairi, Shadia Mohammed Alharbi, Saiful Izwan Abd Razak

**Affiliations:** 1 Department of Chemistry, College of Science, King Faisal University, Al-Ahsa 31982, Saudi Arabia; walarjan@kfu.edu.sa (W.S.A.-A.); halmutairi@kfu.edu.sa (H.H.A.); smalharbi@kfu.edu.sa (S.M.A.); 2 Biomedical Research Center, Qatar University, Doha 2713, Qatar; 3 Department of Mechanical and Industrial Engineering, Qatar University, Doha 2713, Qatar; 4 BioInspired Device and Tissue Engineering Research Group, School of Biomedical Engineering and Health Sciences, Universiti Teknologi Malaysia, Johor Bahru 81300, Johor, Malaysia; saifulizwan@utm.my; 5 Centre of Advanced Composite Materials, Faculty of Engineering, Universiti Teknologi Malaysia, Johor Bahru 81300, Johor, Malaysia

**Keywords:** antibacterial, biopolymers, curcumin, drug delivery, hydrogels, pH-responsive, skin wound healing

## Abstract

Polymeric materials have been essential biomaterials to develop hydrogels as wound dressings for sustained drug delivery and chronic wound healing. The microenvironment for wound healing is created by biocompatibility, bioactivity, and physicochemical behavior. Moreover, a bacterial infection often causes the healing process. The bacterial cellulose (BC) was functionalized using graphene oxide (GO) by hydrothermal method to have bacterial cellulose-functionalized-Graphene oxide (BC-*f*-GO). A simple blending method was used to crosslink BC-*f*-GO with polyvinyl alcohol (PVA) by tetraethyl orthosilicate (TEOS) as a crosslinker. The structural, morphological, wetting, and mechanical tests were conducted using Fourier-transform infrared spectroscopy (FTIR), Scanning electron microscope (SEM), water contact angle, and a Universal testing machine (UTM). The release of Silver-sulphadiazine and drug release kinetics were studied at various pH levels and using different mathematical models (zero-order, first-order, Higuchi, Hixson, Korsmeyer–Peppas, and Baker–Lonsdale). The antibacterial properties were conducted against Gram-positive and Gram-negative severe infection-causing pathogens. These composite hydrogels presented potential anticancer activities against the U87 cell line by an increased GO amount. The result findings show that these composite hydrogels have physical-mechanical and inherent antimicrobial properties and controlled drug release, making them an ideal approach for skin wound healing. As a result, these hydrogels were discovered to be an ideal biomaterial for skin wound healing.

## 1. Introduction

A wound is a type of injury caused by surgery, trauma, infection, diabetes, or other factors. Chronic wounds on the skin have become a life-threatening issue in recent years. According to reports, approximately 10 million people experience burning worldwide, leading to death if not handled properly [1]. Skin wounds of this nature necessitate immediate attention and treatment. Dehydration is caused by burning, which can lead to fatal complications. Choosing a suitable wound dressing is critical in caring for and the treatment of burn and chronic wounds. The material choice is crucial in facilitating quick wound healing and retaining fluidic contents with minimal dehydration [2,3]. An ideal wound dressing should have enough mechanical stability to support skin injuries, flexibility, water retention, antibacterial properties, and tear resistance, and be easily removable. It should also absorb a large amount of wound exudate from the wound surface, as the exudate volume in burning wounds is usually relatively high [4,5]. It should protect against pathogens that cause severe disease by preventing their growth in the wound environment and surface. It should be biodegradable and easily removable to avoid skin peeling after application. Long-term drug delivery and growth factors should be easily swellable with controlled biodegradation. Various advanced wound dressings are commercially available for the care and treatment of skin-burning wounds. Ointment, cream, gels, natural oils, mists, gauzes, and bandages are among the wound dressings available [6,7]. Most chronic wound healing products are effective in wound healing, but their role in skin burns has yet to be determined.

Mechanical strength, swelling, water retention, and controlled therapeutic agent release are all problems with biomaterials. In addition, some of them are poor blood coagulators, while others lack antibacterial properties [8,9,10]. The non-biodegradability of these wound dressings is a significant issue, and most of the materials used in these dressings are also expensive. Taking all of this into account, developing an advanced dressing material with all of the necessary wound-healing and tissue engineering properties [11,12]. Hydrogel-based wound healing materials with all of the above properties can treat chronic and burn wounds. Hydrogels are typically made up of natural or synthetic polymers. However, they lack mechanical properties, biodegradation, and a low swelling ratio, limiting their clinical use as chronic wound dressings [13,14].

It is essential to develop composite hydrogels with enhanced functionalities to address these limitations. Bacterial nanocellulose is a biopolymer and polysaccharide that can retain enough biofluid. Water absorption, swelling, biodegradation, and long-term drug delivery properties make it a good candidate for biomedical applications. Cellulose is the most abundantly available natural polymer and is extensively used in several biomedical applications [15,16]. Graphene oxide is a potential material well-known due to its multifunctional behavior, and it has become popular with researchers due to its potential and versatility, including large surface area, π-π stacking, etc. The most widely credited material for developing next-generation biomaterials is graphene oxide (GO). On the basal plane and at the edge, GO has both hydrophobic and hydrophilic parts, with oxygen-containing functional groups like hydroxyl, epoxy, carbonyl, and carboxyl groups [17]. In addition, GO contains several oxygen-containing functional groups in biomedical applications and essential properties. Furthermore, GO-based materials have multifunctional properties [18].

We have reported the fabrication of composite hydrogels for burn and chronic wound healing applications. The bacterial nanocellulose and different graphene oxide amounts were functionalized via the hydrothermal method. Then, BC-*f*-GO was crosslinked with PVA using TEOS as a crosslinker to fabricate composite hydrogel. According to the best of our knowledge, these formulations have never been reported. The curcumin has been loaded into the composite hydrogel to determine its release behavior and kinetics. FTIR, SEM, water contact angle, and UTM determined the structural, morphological, wetting, and mechanical properties. The swelling was analyzed at different pH in PBS and aqueous media. The in vitro biodegradation and drug release analysis was conducted in PBS media. Antimicrobial were performed against Gram-positive and Gram-negative infection-causing pathogens to observe their antibacterial performance in burn and chronic wound healing.

## 2. Materials and Methods

### 2.1. Materials

A well-reported method was used to synthesize bacterial nanocellulose by a method by Saiful et al. [19], polyvinyl alcohol (PVA), Graphene oxide (CAS No. 763713-1G) tetraethyl orthosilicate (CAS No. 78-10-4), potassium persulfate (initiator), hydrochloric acid (CAS No. 7647-01-0), phosphate buffer saline (MDL No. 806552-1L) solution, and Sigma-Aldrich, Petaling Jaya, Selangor, Malaysia, supplied the ethanol. These chemicals were analytically graded and used as received.

### 2.2. Hydrogel Fabrication

**Scheme 1:**
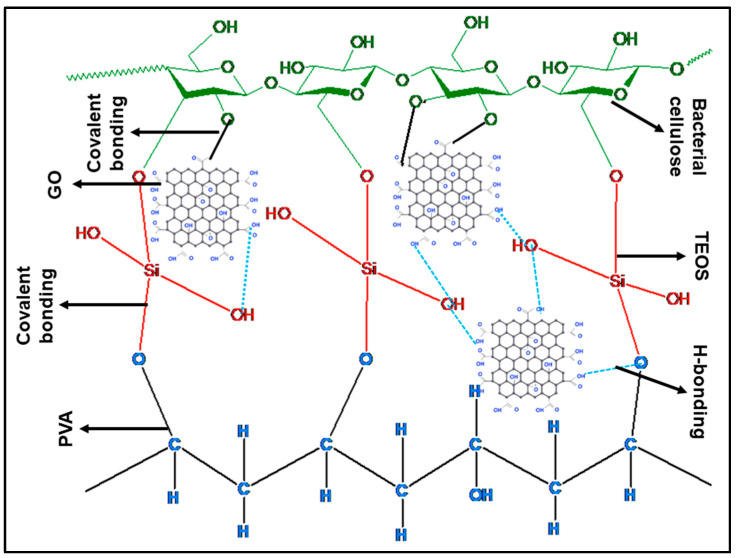
The proposed chemical interaction of the bacterial cellulose, polyvinyl alcohol, GO, and crosslinked via TEOS.

The GO-functionalized-bacterial cellulose (GO-*f*-BC) was synthesized by the hydrothermal approach, in which bacterial cellulose (0.7 g) and various amounts of GO (0.01, 0.02, 0.03, and 0.04 mg) were placed in a 50 mL stainless-steel autoclave for 30 min. The stainless-steel autoclave was placed in the oven at 50 °C overnight to have BC-*f*-GO, and the stainless-steel autoclave was removed after the overnight reaction to obtain BC-*f*-GO, which was then added in 25 mL double deionized water (DDW) and stirred at 55 °C for 1 h to have a homogenized mixture. PVA (0.3 g) was added in 10 mL DDW and dissolved at 80 °C. These were mixed and stirred at 55 °C for 1 h to have a homogeneous suspension. The crosslinker (TEOS (240 μL)) was added to 5 mL ethanol, added into the whorl of the mixture solution, and allowed to stir at 55 °C for another 2 h. Finally, 0.2 g of Potassium persulfate as initiator was dissolved into 5 mL of DDW, added to the solution as an initiator, and stirred at 55 °C for 3 h for successful crosslinking. After 3 h, we had a light yellowish color of the fabrication composite hydrogels and shifted the composite hydrogels into Petri plates. These Petri plates were oven-dried at 45 °C, and different codes (BSG-1, BSG-2, BSG-3, and BSG-4) were assigned to these composite hydrogels after a different GO amount (0.01, 0.02, 0.03, and 0.04 mg). The proposed chemical schematic of the composite hydrogel has shown in Scheme 1.

## 3. Characterizations

### 3.1. FT-IR

The structural and functional group information was analyzed by Fourier transform infrared (FT-IR) Nicolet 5700, Waltham, MA, USA, spectrophotometer. FT-IR analysis was carried out at attenuated total reflectance (ART) mode with wavenumber (4000 to 400 cm^−1^) and 120 scans per sample.

### 3.2. SEM

The surface morphologies of well-dried hydrogels were examined (JSM-6701S, JEOL, Peabody, MA, USA). First, double-sided carbon tape was used to secure the hydrogels to the aluminum stubs, which were then gold-sputtered. Then, micrographs were taken to examine their morphologies.

### 3.3. Water Contact Angle

The wetting behavior of composite hydrogels was studied using a water contact angle system (JY-82, Dingsheng, Chengde, China) to determine their hydrophilicity and hydrophobicity. We recorded wetting at zero and ten seconds to investigate wetting behavior over time.

### 3.4. Swelling

Swell tests were performed in PBS and aqueous media at various pH levels to determine their pH-responsive behavior. The sliced hydrogels were weighed (50 mg), and the initial weight (*W_i_*) was taken. These hydrogels were submerged in PBS and aqueous media-containing beakers at room temperature. Hydrogels that had swelled were removed from the corresponding media. After 12 h, the extra surface media was carefully removed and weighed (*W_f_*) as the final weight and swelling (%) were calculated using Equation (1).

(1) $$Swelling\;\left(\%\right)=\frac{W_{f}-W_{i}}{W_{i}}\times 100$$

whereas: *W_f_* = Hydrogel final weight, *W_i_* = Hydrogel initial weight.

### 3.5. Biodegradation

The hydrogels were tested in vitro in PBS buffer solution (pH 7.4, at 37 °C with 5% CO_2_) to see how they degraded. The squarely sliced hydrogels were carefully weighed (45 mg) and placed in PBS buffer solution to determine biodegradation behavior. Equation (2) was used to calculate the percentage of biodegradation.

(2) $$Weight\;loss\;\left(\%\right)=\frac{W_{i}-W_{t}}{W_{i}}\times 100$$

whereas: *W_t_* = Hydrogel weight at “*t*”, *W_i_* = Hydrogel initial weight.

### 3.6. Curcumin Loading and Franz Diffusion Release

The dermatologic antibiotic curcumin prevents infection in partial-thickness and full-thickness chronic and burns wounds. However, it is still commonly used to treat third-degree burns. Curcumin (10 mg) was dissolved in ethanol (5 mL) and dropped into the polymeric mixture dropwise (BC 0.7 g, PVA 0.3 g, and GO 0.4 mg). The heterogeneous mixture was stirred for two hours at 55 °C, then crosslinked with TEOS (240 µL), and stirred for three hours at the same temperature. In vitro drug release was measured using the Franz diffusion transdermal method at three different pH levels (6.4, 7.4, and 8.4), as Saiful et al. [15] reported in PBS buffer solution at 37 °C. In addition, the calibration analysis was conducted to evaluate the release of Silver-sulfadiazine from hydrogel at different pH levels.

### 3.7. Drug Release Kinetics

We used mathematical models to study drug release mechanisms (3–8) (zero order, first order, Higuchi, Hixson–Crowell, Korsmeyer–Peppas, and Baker–Lonsdale). In addition, data from Franz diffusion at various pH levels were used to evaluate drug release kinetics.

(3) $$\mathrm{Zero-order}\;\;M_{t}=M_{o}+K_{o}t$$

(4) $$First\;order\;\;\;logC=logC_{o}-\frac{kt}{2.303}$$

(5) $$Higuchi\;model\;\;ft=Q=K_{H}\times t^{1/2}$$

(6) $$Hixson\;Crowell\;model\;\;W_{o}{}^{1/3}-W_{t}{}^{1/3}=kt$$

(7) $$Korsmeye\mathrm{r-P}eppas\;model\;\;ln\frac{M_{t}}{M_{o}}=n\;lnt+lnK$$

(8) $$Bake\mathrm{r-L}onsdale\;model\;\;F_{t}=\frac{3}{2}\left[1-{\left(1-\frac{M_{t}}{M_{o}}\right)}^{\frac{2}{3}}\right]\frac{M_{t}}{M_{o}}=K{\left(t\right)}^{0.5}$$

*M_t_* = amount of drug release at “*t*”, and *K_H_*, *K*, and *K_o_* are constants.

### 3.8. In Vitro Analysis

#### 3.8.1. Antimicrobial Activities

The disc diffusion method has been used to evaluate the antibacterial activities of the composite hydrogels, which have been assessed against Gram-positive (*Staphylococcus aureus* (*S. aureus*)) and Gram-negative (*Escherichia* *coli* (*E. coli*) and *Pseudomonas aeruginosa* (*P. aeruginosa*)) bacterial strains. The bacterial strains were provided by ATCC, USA. The molten agar was poured on the Petri dishes and allowed to cool at ambient temperature and bacterial media was applied via cotton swab. Then, the hydrogels (70 μL) were placed on bacterial cultured Petri dishes using a micropipette. The Petri dishes were incubated at 37 °C for 24 h, and the diameter of zone inhibition was measured in millimeters (mm).

#### 3.8.2. Anticancer Analysis

The cytotoxicity of the materials was investigated against the U87 cell line by MTT assay to determine cell viability and cell morphology under in vitro conditions. The purpose of this study is to determine the anticancer behavior of composite hydrogels and drug-loaded composite hydrogels [20].

### 3.9. Statistical Analysis

SPSS Statistics 21 (IBM SPSS Statistics 21, SPSS Inc., New York, NY, USA) analyzed the triplicate data and presented it as mean ± standard error. Figures with Y-error bars represent the standard error. *p* < 0.05, n = 3.

## 4. Results and Discussions

### 4.1. FTIR Analysis

**Figure 1:**
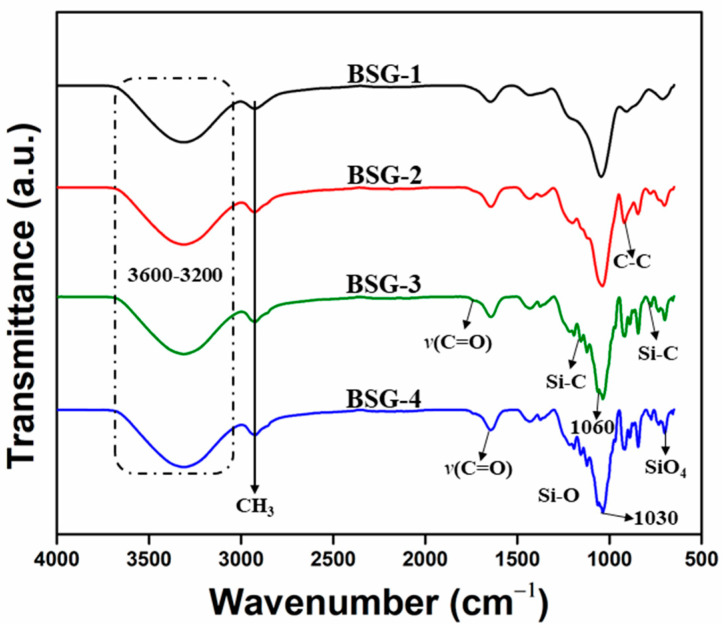
FTIR spectra of composite hydrogels to determine the structural, and functional groups and their physicochemical interactions.

The FT-IT spectral profile of the hydrogel can, as shown in Figure 1, determine the structural and functional analysis of the material present in hydrogels. The vibration band from 1110 to 1000 cm^−1^ is attributed to the asymmetric stretching of –Si–O–C and –Si–O–Si due to TEOS and confirmed the successful crosslinking of bacterial cellulose and polyvinyl alcohol. These polymers and GO were also crosslinked due to hydrogen bonding (H–bonding) that presents the absence peak at 1759 cm^−1^ and broadband at 3600–3200 cm^−^^1^. The increased broadband valley is due to increased intra and inter-hydrogen bonding [21]. The characteristic peaks of BC are hydroxyl, COO^−^, and pyranose rings. The absorption peak of the saccharine structure and pyranose ring is confirmed at 1060 cm^−^^1^ and 876 cm^−1^, respectively. The stretching peak at 2950 cm^−1^ is due to alkyl –CH of BC. The stretching peaks at 1643 and 1469 cm^−^^1^ are attributed to functional groups of C=O and C–C and confirm the presence of GO and it is associated with the polymeric matrix via H-bonding. Hence, the FT-IR spectral confirms successful crosslinking of the polymers and GO interaction with the polymeric matrix that has been determined via available functional groups and interaction.

### 4.2. SEM Morphology

**Figure 2:**
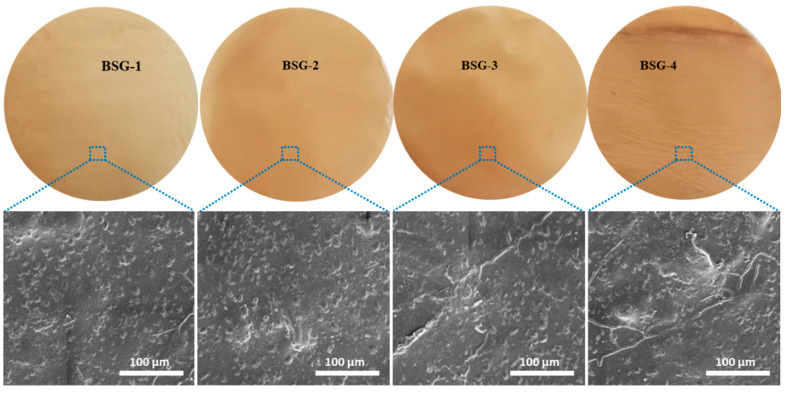
The surface morphology of the hydrogels via SEM.

The surface of hydrogel is a fundamental phenomenon for drug release and interaction with the body’s biological system. Therefore, SEM analysis was performed to investigate the surface properties of the hydrogel materials, as shown in Figure 2. The increasing amount of GO causes more particulate-like (GO-flakes) morphology than smooth surface morphology. These GO-flakes impart their unique role in the hydrogels’ morphology by increasing surface roughness and closing the packing of the hydrogel. Such surface morphology helps burn and chronic wounds by providing them with important hydration [22,23]. However, it was also observed that an increasing GO amount also causes cracking on drying. Hence, it is essential to introduce the only optimized amount of GO to have the desired surface morphology with structural integrity.

### 4.3. Water Contact Angle

**Figure 3:**
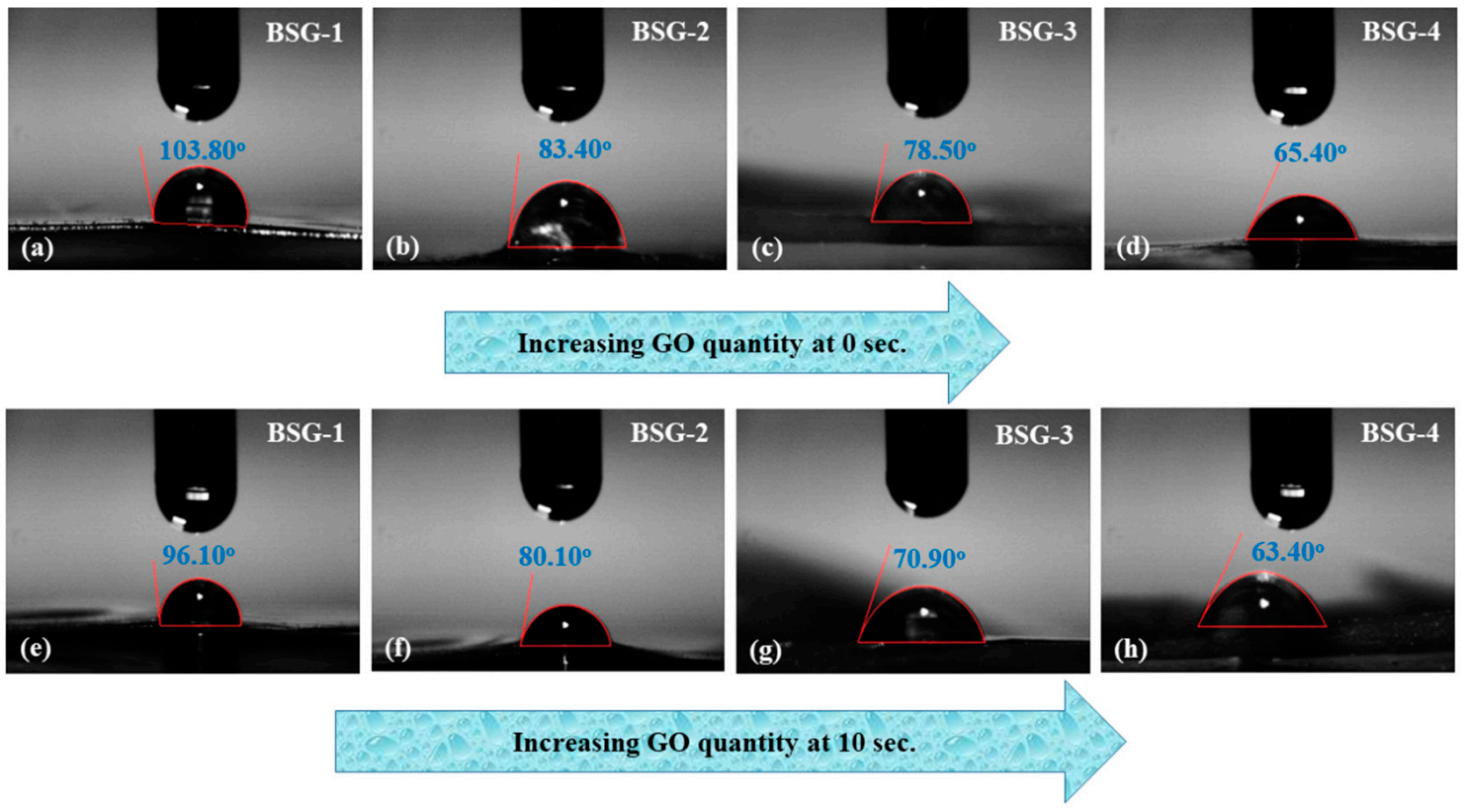
The wetting analysis of the hydrogel was conducted via water contact angle to determine the hydrophilicity and hydrophobicity nature of the hydrogels. The wetting behavior was investigated at different time intervals and 4(**a**–**d**) wetting behavior at 0 s while 4(**e**–**h**) wetting behavior of hydrogels at 10 s.

The ability of a liquid to contact the solid surface is known as wetting. When the liquid and solid surfaces are brought together, they result from intermolecular interactions. A force balance between adhesive and cohesive forces determines the degree of wetting. Wettability is essential in the bonding or adherence of two different materials. Wettability describes the material hydrophilicity and hydrophobicity caused by the surface forces that control wetting. A liquid drop spreads across the surface due to adhesive forces between the liquid and the solid. Due to cohesive forces within the liquid, the drop balls up and avoid contact with the surface [24]. We have observed the wetting behavior of the hydrogels at different times (zero and ten seconds) and with an increased amount of GO. As time and GO amount increase, the hydrogel’s wetting behavior shifts from hydrophobicity to hydrophilicity. BSG-4 and BSG-1 had water contact angles of 103.8° and 65.4° at zero seconds, respectively. However, after ten seconds, the water contact angles for BSG-4 and BSG-1 were 63.40° and 96.10°, respectively. The wetting of BSG-2 = 75.50° and BSG-3 = 83.40° was observed at zero seconds as shown in Figure 3. The wetting analysis clearly shows that the hydrophilicity to hydrophobicity ratio has shifted. That is due to the increasing amount of GO, as it contains several oxygen-based functional groups that caused hydrogen bonding and other weak interactions [25]. A more hydrophilic nature for BSG-4 was observed with increased hydrophilicity by an increasing GO amount and prolonged contact time. Hence, BSG-4 will be helpful to absorb more wound exudate and keep the burn and chronic wound hydrated.

### 4.4. Swelling

**Figure 4:**
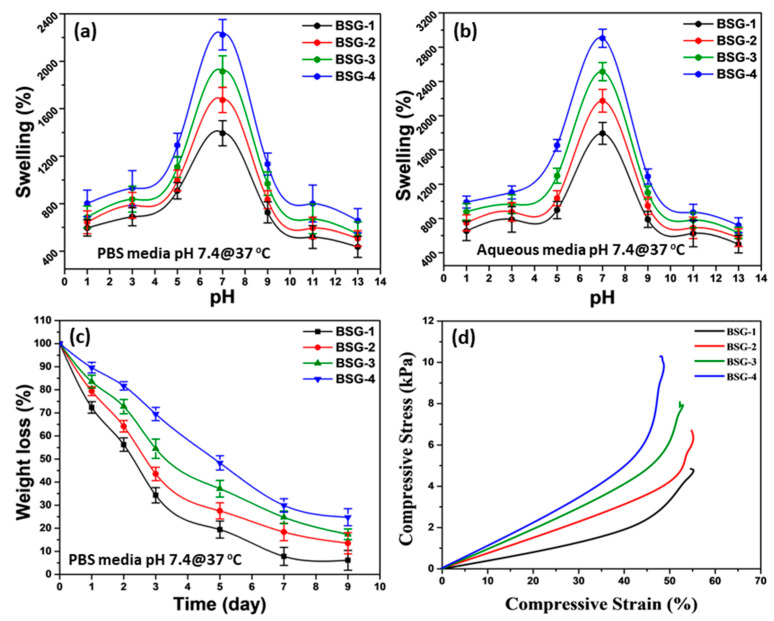
The physicochemical analysis of the hydrogels was conducted to study to analyze the behavior of the hydrogel with biofluid during application. Swelling in PBS (**a**) and aqueous (**b**) media, (**c**) biodegradation in PBS buffer solution (**d**), and stress–strain curve of hydrogels.

Swelling is a significant feature of hydrogels for burn and chronic wound healing and drug delivery. The impacts of pH on the swelling behavior of hydrogels were studied at various pH levels, as shown in Figure 4a,b. All hydrogels showed minimal swelling in acidic and basic pH, with amazing swelling at pH 7. It reveals that hydrogels were extremely sensitive to changes in pH. The hydrophilic groups on the bacterial cellulose, PVA, and GO structures caused swelling due to H-bonding. The water penetrates the void space inside the hydrogels in neutral media. Functional groups in BC, such as carboxyl groups, were protonated to produce more electrostatic repulsion forces [26,27]. The available oxygen-based functional facilitate hydrogen bonding. However, in acidic media, the –OH functional groups have protonated those decreases in swelling, dissociating at higher pH. The hydrogels also have different swelling due to a variable amount of GO. It may act as a crosslinker as it has such a closely packed hydrogel structure due to the enhanced weak intra-hydrogel bonding and electrostatic interactions [28,29]. BSG-1 has maximum, and BSG-4 has minimum swelling in aqueous and PBS media. At pH 7, electrostatic repulsion causes hydrogen bonding and void spaces in hydrogels, resulting in maximum swelling. As a result, the tested hydrogels cause the hydrogel to swell dramatically.

### 4.5. Biodegradation

Biodegradation is a characteristic of drug delivery hydrogels that are likely related to drug release. In vitro degradation of hydrogels was carried out under in vitro conditions in PBS buffer solution. The hydrogels exhibited different biodegradation due to different GO amounts, as shown in Figure 4c. The hydrogel BSG-1 has maximum weight loss, and BSG-4 has the most minor. It may be due to the increasing amount of GO that acts as a crosslinker to hold the polymeric chain tightly. Biodegrading also occurs due to alkyl linkage and glycosidic bonds in BC, facilitating drug release [30]. The different biodegradation also confirms the successful hydrogel fabrication, and the difference in degradation ratio was due to varying amounts of GO. The increased GO amount provides a closely packed structure with increased crosslinking density. The available alkyl linkage and glycosidic bonds of BC breakdown cause biodegradation [31]. Hence, controlled swelling facilitates controlled drug release and other therapeutic agents essential for wound healing.

### 4.6. Mechanical Testing

The mechanical behavior of hydrogels was studied using a stress–strain curve. The mechanical properties of hydrogels provide structural integrity and substantial strength to resist swelling and degradation. The mechanical properties of hydrogels can be controlled by optimizing crosslinker and filler (GO) amounts. It is worth mentioning that increasing the GO amount improved the tensile strength and elastic modulus from BSG-1 to BSG-4, as shown in Figure 4d. Hence, the mechanical strength confirms that our hydrogels have been successfully fabricated with sufficient strength to resist swelling after absorbing wound exudate.

### 4.7. In Vitro Drug Release

**Figure 5:**
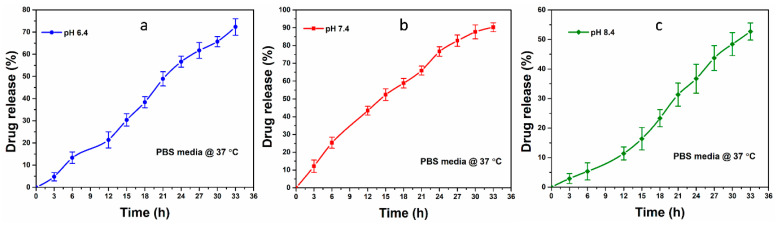
The drug release of curcumin was studied at 37 °C under different pH levels to determine the release analysis behavior: (**a**) Curcumin release at pH 6.4; (**b**) Curcumin release at pH 7.4 and; (**c**) Curcumin release at pH 6.4.

**Figure 6:**
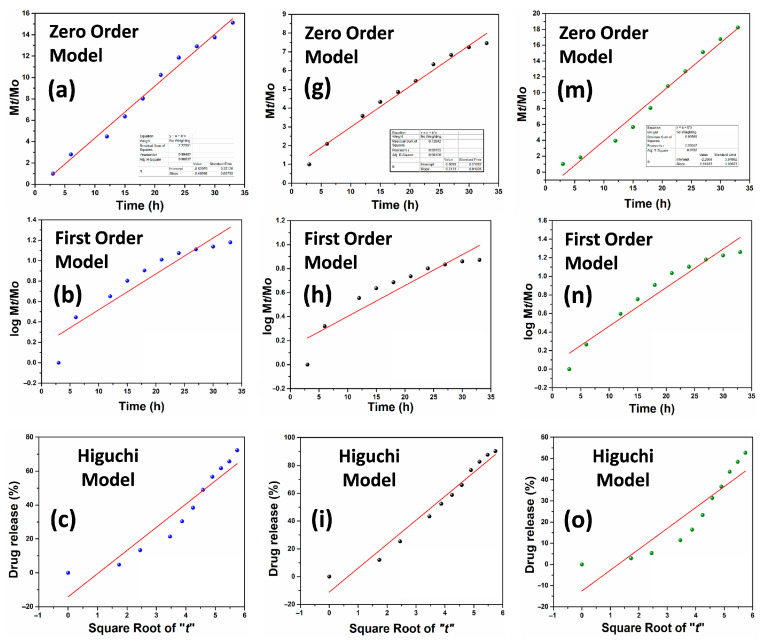

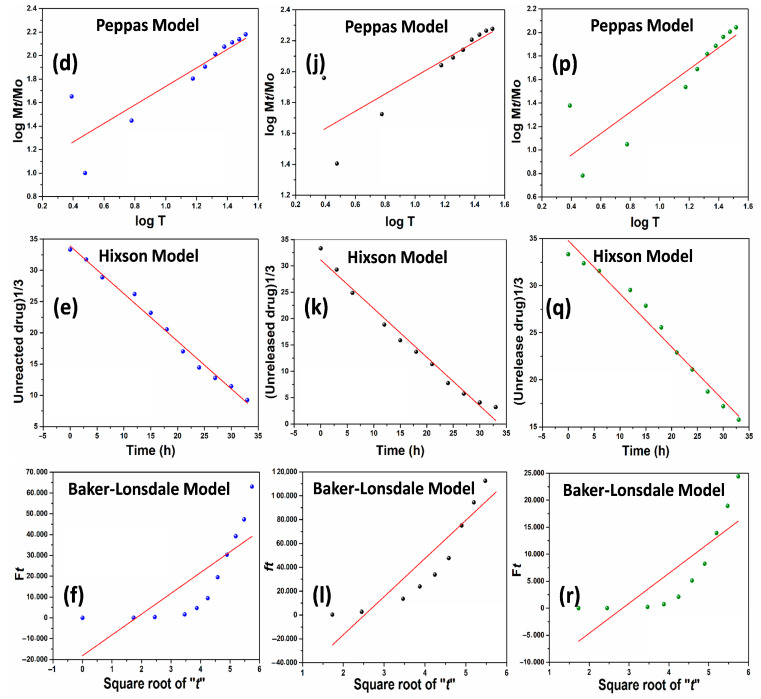
The hydrogel sample BSG-3 was taken to on a random basis to determine curcumin release kinetics at different pH levels (6.4 (**a**–**f**), 7.4 (**g**–**l**), and 8.4 (**m**–**r**)) against different mathematical models (Zero order, First order, Higuchi, Korsmeyer-Peppas, Hixson & Bakers-Lonsdale).

Stimulated release, degradation-controlled release, solvent-controlled release, and diffusion-controlled release are the four mechanisms of drugs released from the polymer network of hydrogels. Solvent-controlled mechanisms include osmotic pressure-controlled and swelling-controlled mechanisms. The swelling of hydrogels is an essential factor in their structural design. It is strongly associated with the controlled release of drugs from hydrogels. The extracellular matrix of hydrogel expands on swelling and makes the drug available on the surface via diffusion pathways. We have taken BSG-4 to load curcumin, which is a natural antibacterial and anticancer drug, due to its optimum physicochemical characterizations. In vitro drug release of curcumin-BSG-4 loaded was determined via the Franz diffusion method under different pH-level (6.4, 7.4, and 8.4) in PBS media, and the drug release profile has been shown in Figure 5. The hydrogels are pH-responsive and offer other drug release mechanisms under different pH levels. The drug release mechanism was found in 7.4 > 6.4 > 8.4 pH, and it is strongly related to swelling and biodegradation of hydrogel, as shown in Figure 6a–c. The pore size of hydrogels increases during swelling due to the diffusion process and the drug is released [32]. Hence, maximum drug release was found at 7.4 pH, and the residual drug could not be determined as the hydrogel films broke into tiny pieces. The drug release can be prolonged by increasing the crosslinker amount. The increasing crosslinker amount can control swelling and degradation, essential factors for the drug delivery system [33]. The drug release at different pH is strongly linked to the hydrogels’ swelling behavior that facilitates drug release on swelling under various other mechanisms. From the drug release profile, a smooth drug release was observed ≈ 91.38% at pH 7.4 after 33 h as seen in Figure 6b. The drug contains hydrogel broken down into pieces and it is not easy to determine drug release from small pieces. The controlled drug release time can be increased or decreased purely depending on crosslinking factors, which optimizes the swelling and degradation of the hydrogels that are necessary parameters for controlled drug release. Hence, these hydrogels could be desired dressing materials with pH-sensitive behavior that could be helpful in controlled drug delivery for burn and chronic wound healing.

### 4.8. Drug Release Kinetics

Drug release provides a deep understanding of the mass transport mechanisms involved. A therapeutic system must provide a specific drug release profile to mathematically calculate the resulting drug release kinetics. Various mathematical models were used to develop simple and complex drug delivery systems and predict overall release behavior. They make it possible to measure an extensive range of vital parameters and apply a model fitting to the experimental release data of curcumin from BSG-4 as presented in Figure 6. Understanding how to use these equations is crucial to grasping the various factors influencing drug release velocity. Their dissolution behaviors affect the efficacy of a patient’s therapeutic regimen [34,35]. These mathematical models have different values of the regression coefficient (*R*^2^) at different pH levels. All the different parameters have been summarized in Table 1, these values provide drug release behavior through the polymeric matrix. Among all the factors, *R*^2^ is the most prominent factor that describes the drug release behavior from different therapeutic systems. The release of curcumin follows various mechanisms at different pH levels that can be described based on the maximum of *R*^2^. We have calculated the value of *R*^2^ against all fitting models. It was observed that at pH 6.4, curcumin follows the Hixson model with a maximum *R*^2^ value (0.99054). The Hixson model describes the release of the curcumin dissolution approach. The release of curcumin was observed to follow zero-order at 7.4 pH by *R*^2^ value (098106). The zero-order describes the quick release of therapeutic agents that is very important to control the attack of the pathogens. However, the curcumin release behavior was observed at pH 8.4 to follow zero-order due to the maximum *R*^2^ value (0.9782). The release of curcumin from the composite hydrogel at pH 8.4 is found to be as effective as it is at pH 7.4. However, the mathematical fitting models have been summarized in Table 1.

### 4.9. Antimicrobial Activities

**Figure 7:**
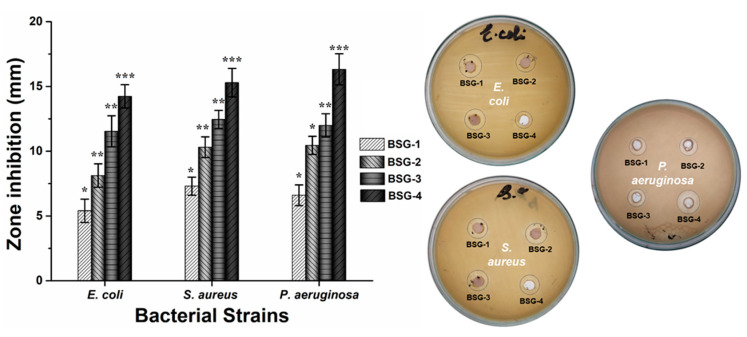
The antibacterial activities of composite hydrogels against different severe skin infections causing Gram-positive and Gram-negative pathogens. * *p* < 0.05, ** *p* < 0.01 and *** *p* < 0.001 and n = 3.

Chronic and burn wounds, and many surgical procedures are all susceptible to bacterial infection. It can lead to severe disease, muscle tissue death, septicemia, and even death. Antimicrobial agents that are effective in treating bacteria are desperately needed. The prepared hydrogels were evaluated against severe wound infection-causing microbes (*S. aureus*, *E. coli*, and *P. aeruginosa*) via the disc diffusion method. The prepared hydrogels were applied to the bacterial lawns in Petri dishes, and these plates were kept in incubation for 12 h. Antibacterial activities were determined via measuring zone inhibition, as shown in Figure 7. These hydrogels have different zone inhibitions against treated bacterial strains. The hydrogel BSG-1 performed minimum, and BSG-4 exhibited maximum antibacterial activity. The maximum antibacterial activities of BSG-4 may be due to the maximum GO amount that ruptures the bacterial membrane with sharp edges of GO [9,36]. On the other hand, the bacterial cell membrane is composed of lipopolysaccharides and phospholipids. The polymeric part of the hydrogel may interact with the bacterial membrane to surround and penetrate several available functional groups to hinder bacterial growth [37,38]. The covalently crosslinked hydrogels via TEOS have inter/intra-hydrogen bonding and weak interactions. BSG-4 exhibited maximum antimicrobial activities, which can be polymeric, and GO components of hydrogels that may communicate with DNA to stop bacterial replication. Hence, the prepared hydrogel could be a potential biomaterial with promising antibacterial activities, which is helpful to treat burns and chronic wounds.

### 4.10. Anticancer Activities

**Figure 8:**
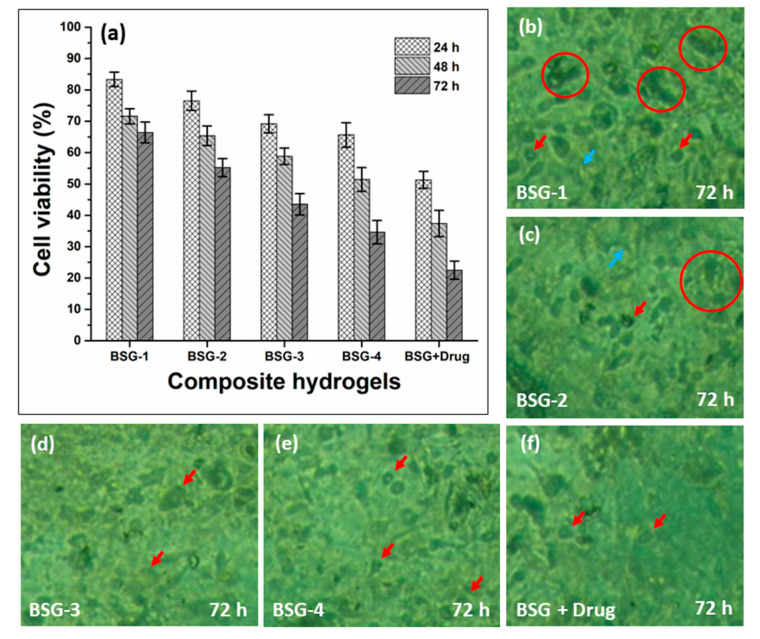
The anticancer activities of composite hydrogels against U87 cell line, (**a**) cell viability in terms of anticancer activities, and (**b**–**f**) cell morphology with different indications.

Figure 8 presents the anticancer activity via cell viability after different time intervals (24, 48, and 72 h) and cell morphology of all samples of composite hydrogels and curcumin-loaded composite hydrogels against U87 cell lines. After 72 h of contact time with composite hydrogels and curcumin-BSG-4 composite hydrogels, nearly 79.56 to 87.58 percent of U87 cells were determined to be nonviable, respectively, as exhibited in Figure 8a. Curcumin combined with PVA/BC-*f*-GO was discovered to have improved anticancer properties. The BSG-4 composite hydrogel also had the highest cell nonviability among the composite hydrogels, which could be owing to the increased GO content [20,39]. The curcumin-loaded-BSG-4 had the strongest antitumor effect. This could be due to the interaction of the composite hydrogel with the cellular membrane, allowing curcumin to conduct anticancer functions. Cell clustering may also be found for BSG-1 and BSG-2, as shown by the red circle, even after 72 h, whereas some cell adherence can be seen for both samples as presented in Figure 8(b–e). However, the red arrows indicated cell detachment, which might occur as a result of cell death, as well as the cell repute. Curcumin was delivered via the drug-loaded BSG-3 composite hydrogel membrane, resulting in increased anticancer activity [36,40]. As a result of the synergetic impact of the release of curcumin-loaded-BSG-3 composite hydrogels after 72 h, the anticancer activities are at their peak. As a result, the curcumin-loaded-BSG-3 composite hydrogel could be used as a biomaterial for wound care and treatment in cancer patients.

## 5. Conclusions

The pH-sensitive composite hydrogels were prepared by blending bacterial nanocellulose with PVA and GO and crosslinked with optimized TEOS. The controlled drug delivery was determined at various pH levels, and the drug release mechanism was investigated using different mathematical models. In hydrogels, FTIR reveals all of the functional groups of bacterial nanocellulose, GO, and TEOS and well-defined crosslinking and hydrogen bonding. GO-flakes were observed via SEM and increasing GO-flakes were observed due to the increasing GO amount. BSG-4 was more stable than other hydrogels. Due to complex polymerization formation, the biodegradation rate of hydrogels was slowed by increasing the GO content. In addition, increasing the GO amount increased the mechanical and hydrophilicity properties. Swelling in the buffer and non-buffer solutions demonstrates that the prepared hydrogels are pH-sensitive. Therefore, hydrogels are a good fit for controlled drug release due to their responsive behavior, and they could be used to release curcumin in a controlled manner. Furthermore, these newly composite hydrogels have exhibited substantial potential, but BSG-4 and curcumin-loaded-BSG-4 have potential antibacterial and anticancer activities. Hence, these composite hydrogels could be promising biomaterials for chronic wound healing applications.

## Figures and Tables

**Table 1:** The drug release kinetics summarizes against different models to determine the best-fitted model at different pH levels (6.4, 7.4, and 8.4).

Drug Release at Different pH-Levels	Models	Intercept/Standard Error	Slop/Standard Error	Regression Coefficient (*R*^2^)
**Drug release kinetics at pH 6.4**	Zero order	−0.52979/0.37126	0.48568/0.01755	0.98837
	First order	0.16701/0.09621	0.03517/0.00455	0.86721
	Higuchi	−14.19939/6.18472	13.67722/1.49206	0.89251
	Korsmeyer-Peppas	0.94986/0.17899	0.78764/0.15055	0.74554
	Hixson	−0.7623/0.2354	33.8974/0.47476	0.99054
	Bakers-Lonsdale	−18,183.23/10,589.06	9974.14/2554.60	0.58753
**Drug release kinetics at pH 7.4**	Zero order	0.8095/0.21262	0.2173/0.01005	098106
	First order	0.14378/0.07845	0.02573/0.00371	0.83965
	Higuchi	−11.10565/4.33	17.22369/1.04596	0.96431
	Korsmeyer-Peppas	1.4071/0.15032	0.56002/0.12644	0.67412
	Hixson	31.14956/0.84	−0.92297/0.04151	0.98014
	Bakers-Lonsdale	−80,632.11/21,021.06	32,004.02/4835.30	0.82628
**Drug release kinetics at pH 8.4**	Zero order	−2.2668/0.65	0.61835/0.03	0.9782
	First order	0.04723/0.08	0.04155/0.003	0.93277
	Higuchi	−12.57362/5.93	9.85459/1.43	0.82256
	Korsmeyer-Peppas	0.59247/0.19	0.91234/0.16	0.76697
	Hixson	34.79252/0.59	−0.56507/0.03	0.97366
	Bakers-Lonsdale	−15,727.59/5918.95	5546.23/1361.49	0.63407

## Data Availability

The data is available in the article.
